# Plant and Animal Genome VIII and Agricultural Microbes Genome I

**DOI:** 10.1002/(SICI)1097-0061(200004)17:1<56::AID-YEA12>3.0.CO;2-H

**Published:** 2000


**Yeast**
*Yeast* 2000; **17**: 56–69.

**Meeting Highlights**

### Plant and Animal Genome VIII and Agricultural Microbes Genome I

http://www.intl-pag.org/pag
http://www.intl-pag.org/agm

### Town and Country Hotel, San Diego, California, USA, January 9–12 and 13–14 2000

**This year was the first time that the agricultural microbe session had split away from the main PAG meeting. However, the meetings were held consecutively, in San Diego, the traditional location for this meeting.**

### Plant and Animal Genome VIII

This meeting was too large for me to cover it in its entirety. Consequently, I have tried to give a brief picture of the status of research in each topic area, highlighting those talks and posters which were most relevant to our readers' interests. I have also featured only those presentations whose subject material fell within the remit of this Journal and have given more weight to those organisms in which significant numbers of comparative and functional genomics studies are under way.

### Animal genomes

#### Aquaculture

This session included reports of several, diverse, fish and marine invertebrate genome studies. Comparative mapping data was presented for the *Fugu* fish genome by Melody Clark (MRC HGMP, UK) and for the zebrafish genome by Angel Amores (University of Oregon, USA). An analysis of the expression levels of several genes in the muscles of catfish has been performed (Kim *et al.*).

#### Poultry

Mapping of the chicken genome is under way and work on the turkey genome map has been started. These maps are mainly used for the placement of QTL for economically important traits. A pilot study of using chicken EST primers on a range of avian species indicated that this method could aid the mapping of these species. Large-scale comparative mapping of the human and chicken genomes and Zoo-FISH of chicken chromosomes using a human chromosome 4 probe were also reported; this early data suggests that there may be a surprisingly high level of conservation of gene order.

#### Swine

The mapping of quantitative trait loci was very much to the fore, in particular that of loci affecting body composition. Several comparative mapping studies between the pig and human genomes were reported and one study comparing pig unigene ESTs to human, mouse and rat unigene ESTs provided yet more tools for these maps.

#### Cattle/sheep

There has been extensive mapping of the cattle genome and a first generation comparative map of the cattle and human genomes was presented. Bob Collier (University of Arizona, USA) presented a novel study utilizing a human cDNA microarray to assess gene expression levels in bovine mammary glands, which identified 1500 genes associated with milk production. A pilot study using chicken ESTs to obtain markers for goat genome mapping may prove useful upon expansion.

#### Equine genomes

Ernest Bailey (University of Kentucky, USA) reported on the status of the horse genome project, which although behind those of the other livestock has made significant progress,

Copyright © 2000 John Wiley & Sons, Ltd.

with linkage maps, synteny maps, and a Zoo-FISH map of the horse and donkey chromosomes under development.

#### Other animal genomes

Edward Rubin (Berkeley, USA) gave a well-received talk entitled 'Cashing in on the human genome program: exploiting the mouse' in one of the general sessions. His group has identified a regulator of interleukin gene expression by comparing non-coding regions from an IL gene cluster in human and mouse. In mouse, the sequence appears to increase the percentage of TH2 cells expressing IL genes rather than increasing the level of transcription in each cell; it is not an enhancer or an isolator. This sequence may be responsible for the asthma QTL mapped to the syntenic human region, since it modulates the levels of expression of IL-4 and IL-13, which in turn affect the level of IgE. Deletion studies of a neighbouring region of the mouse genome, followed by complementation using human YAC clones, identified a liver-specific organic cation transporter.

A study using a microarray containing ~7800 rat ESTs to determine which genes show modulation of expression in response to low serum cholesterol and triglyceride levels was presented by John Ledeaux (Monsanto, USA).

#### Animal comparative mapping

Disappointingly, this session did not live up to its title and was used to discuss the mapping of disease resistance genes in four separate species. Only the pig resistance genes appear to have been identified by comparative mapping using data from the mouse.

### Plant genomes

#### Triticeae

The mapping of the wheat, barley and other related grass genomes is still very much under way. However, a large number of comparative mapping studies have been done, since there is significant colinearity between these species. There is also much interest in disease resistance genes. In a separate barley workshop, intended to update the community on the status of genome research and to provide a plan for future efforts, Gary Muehlbauer (University of Minnesota, USA) summarized the approaches and tools available for barley functional genomics studies.

#### Rice

The physical maps of the rice genome are being improved to the point of sequence ready maps and the sequence analysis is under way. Many QTL mapping projects were presented, ranging from those searching for genes involved in disease resistance to those searching for genes affecting cooking quality and aroma in thai jasmine rice. Thomas Tai gave an overview of the efforts of the Dale Bumpers National Rice Research Centre to develop tools for rice functional genomics.

#### Maize

Jeffrey Habben (Pioneer Hi-Bred International) presented a study of the genes involved in **drought response** using glass slide **microarrays**. The team chose to study unpollinated maize ears, 7 days post-stress and 5 days post-recovery. They identified genes that were induced during stress and also genes that were induced during the recovery phase. ~1–4% of their chosen subset of maize genes showed differential expression, which could indicate that between 100 and 1000 genes are involved in this complex response. They now plan to see which genes are expressed at different levels in tolerant and susceptible strains.

Brandon Gaut (Irvine, USA) presented the results of studies into the **evolutionary genomics** of maize. Maize has a diploid genome that has undergone extensive duplication; ~72% of the duplicated genes in maize are known to be single copy in rice. Using a computational approach, he predicts that this ancient doubling of the genome occurred by a process of allopolyploidy, an event that involves hybridization of two species that were derived from a common ancestor, rather than by autopolyploidy, which involves the formation of unreduced gametes within one species. This theory is supported by the observation that the sorghum genome appears to be more similar to one of the ancestors that hybridized to form the polyploid ancestor of modern-day maize. However, this does not entirely explain the size of the maize genome, which is almost four times the size of the sorghum genome. A search for colinearity within the maize genome genetic map generated evidence for paracentric inversions and large-scale translocations that occurred after the duplication of the genome, but also provided

Copyright © 2000 John Wiley & Sons, Ltd.

evidence for triplicated regions on five chromosomes. These large regions could not have survived from the ancient duplication and could be the remains of a later, whole-genome duplication event or the result of individual large-scale duplication events. Estimating the dates of insertions of retrotransposons, by looking at levels of divergence of LTRs, showed that although a small number were inserted around 5 million years ago, the majority were inserted some 3 million years ago in a genome-wide event. This supports the theory that the maize genome duplicated about 3 million years ago, and would account for the current genome size.

#### International Grass Genome Initiative (IGGI)

Beat Keller (Zurich University, Switzerland) reported on the findings of studies of the levels of **synteny between grass genomes**. A small-scale study comparing sorghum and maize showed a higher gene density in sorghum and a much higher level of variation at the microlevel than was expected between these closely related species. In most cases they found that the synteny at the microlevel was less good than was implied by the maps, thus he feels that comparative map-based gene isolation may not be possible for grasses. A comparison of wheat and barley showed very high colinearity between these two genomes. The region chosen for study contains disease resistance genes and is also highly conserved in the rice genome; however, it is duplicated in the *Triticieae*, which complicates matters considerably. This may well turn out to be a common problem and will affect how often comparative mapping can be used as a tool for these genomes. A study of gene density in rice, maize, sorghum, barley and wheat using individual BAC clone sequences, showed sorghum and barley to be the most gene dense, with one gene per 5 kb, closely followed by rice. The larger genomes had surprisingly high gene densities in the regions studied. He is interested to know if this will remain the case as more sequence is generated and is keen to have large regions of sequence to work with, rather than multiple short sequences.

John Bowers (University of Georgia, USA) reported on a **genetic map** covering the 10 linkage groups of the **sorghum** genome, with 2457 loci. The sorghum genome is 750 Mb in size, which is much smaller than that of its close relative, corn. Comparative mapping with the corn genome showed that several regions were duplicated in the corn genome, but the synteny has remained high. His group is now converting the genetic map into a physical map using probes from several species so that it will also serve as a comparative map.

Jo Messing (Rutgers, USA) presented a sequence-level study of **colinearity between maize and sorghum**. In the region studied, his group found that the genes were closer together in sorghum than in maize; mainly this seemed to be due to the increased presence of retrotransposons between the genes in maize genome. In rice these genes were even closer together. They looked at some gene families and saw that in some genomes these products of duplication were still neighbours, but in other genomes one copy had subsequently moved, possibly due to being close to a retrotransposon. Occasionally, there were variations in copy number; this, he feels, is due to further duplication in one lineage after divergence.

Katrien Devos (John Innes Centre, UK) addressed a burning issue when she presented the results of a small-scale study into the usefulness of ***Arabidopsis*** **as a predictor for cereal genomes, in particular, the rice genome**. Prior studies in this area have met with varying levels of success and there is no clear-cut answer. The rice genome sequencing project has only just started and she admitted that this could affect the success rate in such studies. She screened a short contig of *Arabidopsis* BAC sequences against the rice EST database and got a fairly good hit rate. The rice ESTs which were hit were then screened against the AtDB, and only those which hit only the original *Arabidopsis* BACs were retained. When these ESTs were localized on the rice chromosomes, no evidence for colinearity was observed. A study working in the opposite direction yielded better results. She screened the genes from three PAC clones from rice chromosome I and screened them against AtDB. Of the matches with good probability scores, four were from overlapping BACs on *Arabidopsis* chromosome 4. Repeating the BLAST search with the rice genes just against these four *Arabidopsis* BACs raised the number of matching genes in the region to 9. However, the order of the genes in the two genomes was not conserved. As a result of these studies, she feels that there is not significant colinearity between *Arabidopsis* and rice and that the homology at the sequence level is not always high enough to be useful.

Copyright © 2000 John Wiley & Sons, Ltd. 

#### Forage and turfgrass

The work in this field has concentrated on the loliums, with a genome initiative and physical, genetic maps under way. Comparative mapping studies were presented by Toshihiko Yamada (HNAES, Japan), Andrew Patterson (AGTC, USA) and Scott Warnke (Oregon State University, USA).

#### Sugarcane

Genetic and physical maps of the genome are under way. A Brazilian EST programme was announced by William Burnquist (CTC, Brazil) and a survey of the resource to identify disease resistance-related ESTs was presented by Luis Camargo (University of Sao Paolo, Brazil).

Roseanne Casu (CSIRO, Australia) presented preliminary results of a screen for genes involved in sucrose accumulation. Again, since this crop is economically important there is great interest in the mapping of disease resistance genes.

#### Sugarbeet

The study of this genome is at the stage of genetic mapping and definition of chromosome organization. However, gene expression studies applying differential display to the hunt for genes involved in stress response during germination and nematode resistance were presented by Benildo de los Reyes (USDA-ARS, USA) and Michael Kleine (University of Kiel, Germany), respectively.

#### Legumes

The study of the soybean is furthest ahead amongst these species. Genetic mapping studies have divided the genome into linkage groups. David Grant (USDA-ARS, USA) reported on an investigation of soybean synteny with *Arabidopsis*, which produced evidence for genome duplication in *Arabidopsis*. John Erpelding (USDA-ARS, USA) gave a status report for the soybean public EST project, and Lila Vodkin (University of Illinois, USA) announced a functional genomics program for soybean.

#### Cotton

Physical mapping and QTL mapping of the genome is under way, with BAC clone and microsatellite resources under construction.

#### Compositae

Dense maps of the two main lettuce species under study (*L. sativa* and *L. saligna*) have been presented by Rick Kesseli (University of Massachusetts, USA) and mapping of the sunflower genome is also in progress.

#### Solanaceae

Jim Giovannoni (Texas A&M, USA) talked about a multi-institutional programme to provide tools for tomato functional genomics, including the sequencing of many ESTs with the goal of expression profiling studies of fruit development and response to pathogen attack in the tomato. Uwe Wirtz and Barbara Baker (USDA-ARS-PGEC, USA) announced a functional genomics programme for the potato. They plan to sequence 55 000 ESTs and then make an array of selected ESTs for expression analysis studies, and they also aim to identify disease resistance genes and continue studies of the synteny to tomato.

#### Fruit and nuts

Citrus genome maps and EST analyses were reported. A gene expression study of fruit ripening in the strawberry, using chip technology, was presented by Asaph Aharoni (CPRO, The Netherlands). Mapping studies utilizing SNPs in winegrape to identify those causing observed variations between strains, and to identify those involved in metabolism and defence response, were presented by Maria-Teresa Cervera (CSIC, Spain) and Riccardo Velasco (IASMA, Italy), respectively.

#### Ornamentals

A genetic map of the rose genome was presented and the rose was proposed as the model for this group of plants.

#### Forest trees

EST projects were announced for several species and ESTdatabases have been initiated. A comparative map of *Eucalyptus* was reported and a new database, Tree Genes 2000, was announced.

A study of drought response in maritime pine using a proteomics approach was presented by Christophe Plomion (INRA, France). His group ran 2-D gels of pine needles from drought-stressed and control plants. Of the 1000 spots that repeatably showed differential expression, they concentrated on 163. The genes which were induced by the

Copyright © 2000 John Wiley & Sons, Ltd. 

stress included chaperone genes, and those involved in detoxification and carbon metabolism and down-regulated genes included those involved in the lignification process. Mapping of some of these genes showed that they correlated with the three drought QTL loci that the group had identified.

**Brassicas**

A great deal of comparative mapping has been done between *Brassica* genomes and also between *Brassica* genomes and the *Arabidopsis* genome, since they include the crop plants most closely related to *Arabidopsis.*

Ian Bancroft (John Innes Centre) presented the results of a comparative mapping study of *Brassica oleracea* and *Arabidopsis*. His group has constructed a BAC library of the *B. oleracea* genome to aid these studies. They selected two regions that are triplicated in the *B. oleracea* genome and compared those six regions with the two *Arabidopsis* counterparts in terms of gene content, gene order and gene spacing; in each case they found extensive divergence (see also the review, 'Insights into the structural and functional evolution of plant genomes afforded by the nucleotide sequences of chromosomes 2 and 4 of *Arabidopsis thaliana*', in this issue).

Carlos Quiros (University of California at Davis, USA) reported similar findings in a study of the *B. oleracea* homologues of the genes from a small region of chromosome 4 of *Arabidopsis*. As expected, the group showed high homology in the exons, which dropped off significantly in the introns. In addition, the introns were shown to vary in length. There was some conservation in promoter regions, but this varied across the three genes studied. He feels that comparisons can therefore be used for prediction of gene content and structure by homology, but not for studies of genome structure or genomic origin, since these will be complicated by differential duplications in the two genomes.

**Arabidopsis**

After 10 years of the publicly funded genome project, there are now dense genetic and physical maps and a complete BAC map. The genomic sequence is almost complete; two near chromosome sequences have been published so far. Steve Rounsley reported on the analysis of the **near-complete sequence** of the **Landsberg *erecta*** ecotype genome that has produced using a shotgun strategy at Cereon, USA. They have ~95% coverage, as ~50 000 contigs which cover 92 Mb, and singleton clones which cover the remainder of the 265 Mb they have sequenced. 96% of the ~2,200 *Arabidopsis* ESTs in GenBank have a match in the sequence, thus they are confident that almost every *Arabidopsis* gene will be at least partially represented by their sequence. Comparison of their data to the public dataset, produced from the Columbia ecotype, has allowed the detection of SNPs and insertions and deletions, which are mainly due to repeat expansions or variations in the positions of insertion of transposons. The data yielded from this comparison is to be released publicly, including access to the SNP data.

Samir Kaul (TIGR, USA) presented the results of the analysis of the sequence of **chromosome 2**. The sequence was produced by choosing seed BACs and then using end sequencing to extend in both directions. 19.6 Mb of the chromosome is unique, 4 Mb forms the rDNA and the centromere accounts for 1 Mb. The chromosome is predicted to comprise 4116 genes, 4037 of which encode protein products. The mean GC content is 35%, the gene density is 1 gene per 4.4 kb and the mean intron size is 200 bp. The gene density and GC content are uniform across the chromosome, but drop at the centromere. Of the 912 genes with a sequence match to another part of chromosome 2, 449 are in tandem clusters, which can contain up to 10 genes. There are also interchromosomal duplications, a 4.5 Mb region is duplicated on chromosome 4 and a 700 kb region is duplicated on chromosome 1. Sequencing of the centromere boundary BACs yielded ~80 genes, several of which had EST matches and may still be active, hence they argue that efforts should be made to sequence further into the centromeres in all genome projects.

The analysis of the **chromosome 4** sequence was presented by Dick McCombie (CSHL, USA). This chromosome is 17.38 Mb long and represents ~17% of the genome, comprising 3744 protein-encoding genes. The GC content and gene density are the same as for chromosome 2. A condensed heterochromatic region was discovered on the short arm, which harboured a higher frequency of retrotransposons and tandem repeats; furthermore, none of the genes found there had EST matches. The genes predicted from the sequence were assigned functional classes using the FUNCAT software from MIPS. Genes flanking the centromere were found to

Copyright © 2000 John Wiley & Sons, Ltd. 

be less likely to be assigned to a functional class. Surprisingly, genes with unexpected matches were discovered, e.g. one gene had a match to Brca1; unfortunately, this type of match provides no clear indication as to the role of the gene in the plant.

Burkhard Schulz (University of Cologne, Germany) presented a reverse genetics approach to functional studies of *Arabidopsis* genes. **Two-hybrid analyses** of known genes were used to identify their interaction partners. Existing mutant libraries were then screened by PCR to identify mutants of the genes encoding the interaction partners. The genes were then cloned by using expression libraries to rescue the mutant phenotypes. Once the cloned genes were obtained, expression pattern studies could be carried out.

Robert Schaffer (Michigan State University, USA) described a pilot study using a **microarray** with 11 000 non-redundant *Arabidopsis* ESTs, and announced a **public resource** for screening of this array. The ESTs have been obtained from libraries produced from different tissues, at different developmental stages and at different times of day, thus they should be able to represent most conditions in the plant. The pilot study looked for a rare group of genes, the circadian clock-related genes, using samples taken at midnight (time 0) and noon. The expression of most known cycling genes varied as expected, with ratios greater than 2. However, circadian clock genes differ from light responsive genes in that their expression varies with time, irrespective of lighting, so they then used samples at 24 and 36 hours, when the plants were left in the dark at 36 hours. A small proportion of the cycling genes continued to show cyclic expression; these are circadian clock genes. The Michigan State University section of the *Arabidopsis* Functional Genomics Consortium (AFGC) now propose to provide a microarray screening service for the *Arabidopsis* community. They will accept applications three times a year, with an initial restriction of only two experiments per lab. Those interested should go to: **http://afgc.stanford.edu**

A large audience heard Pamela Green (Michigan State University, USA) present the results of research into genetic determinants of **mRNA stability** in *Arabidopsis*. mRNA abundance is a function not only of transcription rate but also of the rate of degradation of the message. Altering the stability of a message can be a quick way of increasing or decreasing mRNA levels. If synthesis ceases, an unstable message is cleared from the cell in minutes, whereas a stable one might take from several hours, even to days to be cleared. Sequence-specific decay and general decay both contribute to the rate of clearance, e.g. premature stops and motifs such as AUUUA cause rapid decay. The DST sequence, which causes messages to be highly unstable, is unique to plants. It has been shown that messages with this motif are cleared $\sim$2 minutes after blocking of mRNA synthesis. Small changes in the DST motif deactivate it, proving that this is sequence-specific degradation. Pamela's group have worked on trying to identify the proteins responsible for degradation of mRNAs in *Arabidopsis*. They have identified two genes which regulate the response to the DST motif, DST1 and DST2. They also looked for *Arabidopsis* homologues of the yeast exoribonuclease *XRN1* and found three genes with significant homology, all of which could complement *xrn1* mutant yeast. They then used microarray analysis to see which messages are targets of each XRN gene by using probes from mutant *Arabidopsis* strains. The microarray had 600 elements, including DST containing genes, highly expressed genes, RNA binding proteins and RNAses. They already knew from Northern analyses that deleting DST1 only makes a two- to three-fold difference in mRNA abundance, and in the absence of genes showing high expression ratios, they looked at genes with the highest expression ratios, between 2 and 5, which would commonly be ignored in other experiments. 11 of the 12 messages that were up- or downregulated by two- to five-fold were confirmed by Northern analysis. This leads them to assume that these are genuine small-scale changes. Those interested should go to: **http://afgc.stanford.edu**

Rob Martienssen talked about the *Arabidopsis* functional genomics studies that are going on at the Cold Spring Harbor labs (CSHL, USA). They are using a **gene-trap technology** to generate mutations in *Arabidopsis* genes. In this system they use an Ac/Ds-based transposon, containing the GUS gene (a plant *lac*Z equivalent) preceded by an intron and splice acceptor sites in all three frames. This allows fusion with any upstream *Arabidopsis* exons once the inserted transposon is induced to jump around the genome. They have generated thousands of lines in this way, which they are currently analysing. The reporter gene shows the expression pattern of the native gene and can give functional information in cases where the insertion causes loss of function. By

Copyright © 2000 John Wiley & Sons, Ltd.

looking for genes with similar expression patterns, they have identified genes whose expression is regulated by a known transcription factor. This was confirmed by showing that these genes lost their highly specific expression pattern when the fusion lines were crossed with one in which the regulator gene was deleted. They plan to systematically mutate every *Arabidopsis* gene and to develop a database to handle the data produced, called GeneTrapDB. For each mutant they aim to sequence the DNA upstream of the insertion, to stain for expression pattern and to perform functional screens. So far they have sequenced over 3000 mutants, finding hits for over 2000 annotated genes. However, 90–95% of the insertions have no obvious phenotype in the tissues in which the gene in question is expressed, a similar problem to that being encountered by yeast and mouse researchers. This could be due to the gene having a conditional phenotype, or to the gene being lethal at an early stage or because of redundancy in the *Arabidopsis* genome (for which there is mounting evidence). They have found clear examples of each case amongst the genes that have been studied so far. Other work includes an investigation into replacing the GUS gene with GFP, which is easier to spot, and an attempt to apply this technology to maize. This project has started with an effort to increase the number of gene sequences available, by making a library enriched for unmethylated coding sequences, so that they are better able to tell which gene has been interrupted by the insertions.

### Technology

Charlie Rodi (Sequenom, USA) described an **'error-free' system for detection of SNPs** and determination of allele frequencies. In this system, the template is amplified and then in a second amplification with gene specific primers, nucleotides are incorporated across the polymorphic site. The products are spotted onto hydrophobic patches arranged in a grid on a 'SpectroCHIP' and analysed by MALDI-TOF mass spectrometry. Alleles are discriminated as peaks and the genotype calling has been automated. The genotype-calling software was trained using 2000 bad spectra (that had been verified by repeating the analysis to get good spectra). In tests, the conservative setting of this software has never called a false SNP and neither has the moderate setting; however, the aggressive setting does make some erroneous calls. This 'error-free' calling is crucial for population studies in which you can need to type as many as three times more samples if you have as much as a 1% calling error. For example, for a known T-A polymorphism, the secondary PCR is performed with a primer close to the polymorphism and with ddATP instead of dATP. This causes the product from alleles with the T form to terminate at the polymorphism and the A form products terminate at the next T. This difference in size is easily detected by MALDI-TOF, which can discriminate products differing by only 2–3 Da in molecular mass, and the system can distinguish a homozygote from a heterozygote in one experiment. A test of the system on pooled samples using a biallelic site in a known gene showed that the system can also detect previously unknown polymorphisms. A third peak was observed that was 25 Da away from one of the pair of expected peaks; this was the difference between incorporation of a T or a G. Sequencing of individuals confirmed that the locus was in fact triallelic. This test also showed that the system can be used on populations to assess whether a site is bi-, tri- or tetra-allelic and to give a rough idea of the frequency of each allele (as yet it is not clear how quantitative the system is). The current throughput of analyses that they have achieved is 20 000 alleles per 4-hour run of the mass spectrometer and the calling software.

Robert Lipshutz (Affymetrix, USA) reported on work aimed at **increasing the throughput of SNP analysis**. To test their system they produced a chip loaded with oligos covering 1500 bi-allelic SNPs. For each SNP there are several oligos containing the A or B allele of the SNP at different positions and also mismatch SNPs to check for false hybridization. They have found that they can perform the PCRs required for the sample prep in 24 pools, without losing repeatability, and they can achieve $\sim$98% accuracy, depending on the stringency of the calling. They can type seven samples per hour per scanner for these 1500 SNPs. They have also produced a standardized tag array with 16 000 tags (Genflex). For any known SNP, e.g. an A/C SNP, the probe PCRs are performed using ddTTP and ddGTP nucleotides that have been labelled with two different fluorescent dyes. These are then hybridized to the standard array and in this way 4000 SNPs can be typed at a time. A computerized system discriminates the signal and

Copyright © 2000 John Wiley & Sons, Ltd. 

this can also be used with pooled samples, without losing discrimination. This system can be used to compare strains and has already been applied to the comparison of the *Arabidopsis thaliana* Landsberg and Columbia ecotypes.

Elizabeth Kerr (Affymetrix, USA) gave a presentation on the **applications of the Affymetrix chips to expression analysis**. Each chip has 200 000 individual probe cells, each with a unique 25-mer oligo which is directly synthesized and linked onto the glass substrate. The cRNA probe is made using biotin-labelled UTP and CTP; after hybridization the chip is washed with labelled streptavidin. Each gene is represented by several overlapping oligos and mismatch oligos, which allows a high certainty for the qualitative score and the assessment of background and the stringency of the hybridization. Users typically achieve a sensitivity of 1 in 100 000, but they have achieved 1 in 300 000 with in house experiments. They have tested the repeatability by hybridizing two chips with the same sample. Only 0.3% of the genes showing a difference between the chips differed by more than two-fold; when they hybridized two chips with probes from two different flasks of the same strain, the difference only rose to just less than 1% of the differences being greater than two-fold. Since each gene is represented by multiple overlapping oligos, these chips can help to deal with gene families and related genes. One or more of the oligos can be designed to lie in regions of poorest homology or in non-shared domains, thereby determining which gene is actually being expressed. These chips have already been applied to cell cycle-regulated expression patterns and to identifying genes which are up- and downregulated in colon cancer. They are currently planning *Arabidopsis* and *Drosophila* chips with approximately 9000 genes and ESTs on each and are looking at making chips for crop plants and microbes such as *Haemophilus influenza* and *Escherichia coli*. They have the whole yeast gene set on four arrays, but are planning to reduce this down to one array. They have 30 000 genes and ESTs on a mouse array set, a set of three arrays for the rat and a set of human arrays.

### Bioinformatics and databases

### Verbal presentations—bioinformatics tools

Jo Dicks (John Innes Centre, UK) presented **Chromtree**, which is a **phylogenetic analysis package** that deduces a phylogenetic tree from chromosomal data such as banding patterns, gene order or segment order, for single chromosomes or sets of chromosomes. There are several mechanisms of chromosomal rearrangement, including translocations, inversions, centric fusions and dissociations. These can result in changed gene order, altered orientation of segments and movement of segments onto other chromosomes. For any given chromosome, combinations of these events have occurred over time in an unknown order. For any two chromosomes there are an infinite number of paths by which one could have been formed from the other. The programme produces a tree of the paths that is pruned so that only rearrangements that make progress towards the goal are accepted. This is then used to assess the evolutionary 'distance' between the two chromosomes (see also the review 'Graphical tools for comparative genome analysis', in this issue).

Ron Taylor (NCI, NIH, USA) presented **a new tool which uses a Bayesian similarity measure to compare microarray results**, which is aimed at determining whether or not two samples were taken from cells at the same cellular state. To perform this analysis, a new metric was derived and the programme (for which the source code is available) was written in Perl on a Sun UNIX workstation. The analysis was tested on all the available yeast data, which took 6 hours. The result was that the new analysis tool assigned two samples which were taken one cell cycle apart, but at the same point in the cell cycle as the closest, whereas the other two existing methods, based on Euclidian and correlational methods, both assigned cell cycle time points which were next to each other as most similar. This could indicate that this new method is better at detecting similar cell states, and Ron feels that the analysis will perform better as more data is generated (a paper describing the programme has been submitted to *Bioinformatics*).

### Verbal presentations—general interest databases

Rolf Apweiler (EBI, UK) gave an overview of **InterPro, a new protein database designed** to replace SwissProt. There are currently 2423 entries in InterPro, representing 1776 protein families and 615 domains. InterPro entries will have links to their Pfam, PRINTS and Prosite entries, in addition to information about the protein or domain. Services provided will include the ability to compare

Copyright © 2000 John Wiley & Sons, Ltd.

entries for different organisms and to do Pfam, PRINTS or Prosite analyses on user sequences, although these will take some time to perform. They are looking into allowing batch queries and improving the time factors of these searches. Rolf explained that one of the main reasons that protein databases are growing at a much slower rate than DNA databases is because the annotation is a major bottleneck in the process of assembly. Consequently, other work is concentrating on automated data collation; however, SwissProt is considered to be their 'gold standard' data, which they do not wish to 'dilute', and so they are using TREMBL to try out automated methods of data entry. Interested parties should consult: **http://www.ebi.ac.uk/interpro/**

Jason Stewart (NCGR, USA) presented the **GeneEx software** that is currently under development. This is a relational database management system with server side analysis tools, which will be available to be uploaded by users, for which source code will be made available. It is planned to be cross-species and able to cope with various technologies, although the main focus so far is the handling of microarray data. Users would be able to store their data securely in a confidential fashion; it would then be released publicly upon publication of the work. Access to the relevant centrally stored data could easily be arranged for all members of each project, irrespective of their geographical location, a feature which should appeal to international consortia. They also plan to mine the currently available organism-specific expression databases and have designed an 'Upload' tool to collect and validate data for the database. They are working with a consortium of researchers looking at gene expression in a variety of organisms to produce a gene expression mark-up language (GEML) and to discuss whether image files should be included. Those interested should look at **http://www.ncgr.org/research/geneX**. Those wishing to participate or to download the currently available tools should send an e-mail to **genex@ncgr.org**

Pedro Mendes (NCGR, USA) spoke about **a new metabolic pathway display tool, PathDB**, that is currently being developed. This tool displays chemical structures in each pathway. The metabolites are treated as nodes and the reactions as edges linking the nodes. They have devised a radial method of displaying pathways, which makes complex and branching pathways much clearer and reduces the amount of crossing lines. They also plan to include compartmentalization data and transport steps and to allow users to use the tool to build their own pathways. One criticism was the lack of indication as to which reactions are reversible; they are working on ways to solve this.

**Verbal presentations—organism-specific databases**

Lois Maltais (The Jackson Laboratory, USA) gave a presentation on the current status of the **mouse genome informatics** available at the Jackson Lab web site. The resource is broken down into five main parts, the mouse genome database (MGD), the gene expression database (GXD), the mouse genome sequence database (MGS), the mouse tumour biology database (MTB) and the rat genome database (RGD). MGD contains genetic, physical, cytogenetic and comparative maps lists of orthologues and information on gene characterization and phenotypes. GXD contains expression data produced by a selection of methods, linked to a hierarchical anatomy listing. MGS is a collaborative effort between five genome centres and contains biologically significant sequence features, which are linked to biological information. MTB has information on tumours observed in the mouse, such as name, classification, incidence, pathology and also images of tumours. It also has links to the genes that have been shown to cause susceptibility or to show expression changes linked to tumour progression. RGD contains information on genetic markers, gene names and locations, polymorphisms and maps and provides links to MGD. The curators are part of a multi-species consortium on nomenclature that is aiming to define a consistent terminology for gene names, etc. The next meeting (INW3) will be held at the 'High Throughput Genome Sequencing' meeting in Breckenridge, Colorado, USA in June 2000.

David Grant (USDA/ARS, USA) spoke about the **Soybase database** for information on the soybean. Work is under way to produce a composite genetical and physical map. Genetic markers are being mapped onto existing BAC clones, which will be end-sequenced. However, the low rate of polymorphism in the genome does pose a problem for genetic mapping. Currently the database contains information on DNA segments, genes, QTLs, proteins and metabolites. There is also a germplasm database and they aim to include the data from cDNA sequencing and gene expression pattern studies.

Copyright © 2000 John Wiley & Sons, Ltd. 

Mary Polacco (USDA/ARS, USA) talked about the efforts of a consortium of plant genome researchers and database curators to produce a **consistent terminology** for such items as traits, phenotypes, tissue names, gene names, enzyme names and expression pattern terms. They want to use this to allow them to make databases compatible, which would allow cross-database searches to be performed.

#### Computer demonstrations

**FLYBASE** has an impressive hierarchy of anatomical data and gene expression pattern data is being integrated with this. The integration of the Berkeley data into FLYBASE is still under way and it does not yet provide certain searches, which they aim to add, such as for specified expression patterns. The curators are interested in hearing from non-fly researchers who use the database, to allow them to ensure that it fulfils the needs of all users: **http://flybase.bio.indiana.edu/**

An object-oriented query system for comparing multiple cereal crop genes, which was written using object protocol model (OPM) tools, was demonstrated using rice and maize genes (OPM tools are available free to academic institutions from Gene Logic Inc). It has been written with compatibility issues in mind, being designed to upload data from multiple sources: **http://mulciber.rnet.missouri.edu/~genelogi/dbs/crops**

There were also demonstrations of databases that are described above, including:

Interpro: **http://www.ebi.ac.uk/interpro/**
MGD: **http://www.informatics.jax.org**
PathDB: **http://www.ncgr.org/software/pathdb/**

### Agricultural Microbes Genome I

Pascale Cossart (Institut Pasteur, France) reported on the preliminary results of the analysis of the **recently completed genome of *Listeria monocytogenes***. This Gram-positive organism is the most virulent food-borne pathogen, with an overall mortality rate of 30%. The 2938 kb genome exists as one circular chromosome, which has been sequenced by a consortium of eight European groups. Amongst the new predicted genes, early analyses have identified 14 new membrane-anchored members and four new secreted members of the internalin family, which are good candidate virulence factors since they are surface proteins. There are non-pathogenic *Listeria*, species such as *L. innocua*, which they are currently working on. They plan to compare the two genomes in an attempt to identify the genes responsible for the pathogenicity of *L. monocytogenes*. The sequence is almost complete and already they have identified regions from the *monocytogenes* genome that are absent from the syntenic regions of the *L. innocua* genome and the closely related *B. subtilis* genome. However, it is not yet clear if the virulence genes were inserted into *innocua*, resulting in *monocytogenes* or if *innocua* is a deleted version of *monocytogenes*. They have as yet found no evidence of pathogenicity islands like those described in some other bacteria.

Frank Kunst (Institut Pasteur, France) talked about the progress that has been made towards the complete sequence of the genome of ***Photorhabdus luminescens***. This organism is a symbiont of a nematode worm and produces antibiotics and fungicides. The antibiotics kill off any competing Gram-positive bacteria, such as *Bacillus subtilis*, and are only expressed by one of the two phase variants; the other variant is non-motile and does not make antibiotics, even though the genes are still there, implying transcriptional regulation of some kind. They currently have 45 000 sequence reads, which correspond to 97% of the 5.5 Mb genome. They have constructed 800 contigs; however, it is difficult to assemble the sequence due to the high level of IS repeat elements. They have already found several operons, including a toxicity operon, which is more similar to *Vibrio cholerae* than to the more closely related *E. coli*. They plan to make comparisons to both Enterobacteriaceae and Entomobacteriaceae and have already found several good matches.

Andrew Simpson (Ludwig Institute, Brazil) proudly announced the completion of the sequencing of the ***Xylella fastidiosa*** genome, **the first plant pathogen to be sequenced**. This Gram-negative pathogen, which attacks citrus fruits, is transmitted by leaf hopper insects. It appears to block the xylem and may produce toxins, causing severe symptoms in the citrus crops essential to the Brazilian economy. The 2.7 Mb genome exists as a circular chromosome and there is also a megaplasmid and a miniplasmid. Twenty-nine laboratories were chosen from 100 applicants across Brazil and the efforts were coordinated via the Brazilian virtual genomics

Copyright © 2000 John Wiley & Sons, Ltd.

institute, ONSA. The analysis of the genome predicted 2750 putative ORFs, of which 39% have no match in the public databases. Retroviruses were detected in the genome, some of which contained plant derived genes. The *Xylella* genome is most like *E. coli* and *H. influenzae*; it has plenty of small molecule biosynthesis and monosaccharide metabolism genes but appears to have different polysaccharide metabolism from *E. coli*. It has a complete set of DNA repair genes, but a lower number and a smaller variety of transporters. They also found genes for adhesin and xanthan gum production, which could cause bacterial aggregation and blockage of the xylem. They hope to move on to functional studies of the predicted genes.

Vivek Kapur (University of Minnesota, USA) described the sequencing of the genome of ***Pasteurella multocida***, a Gram-negative multispecies pathogen, which can attack swine, cattle, humans and birds. There are individual clones that are associated with different geographical regions and with different hosts. His group are sequencing the genome of one of the three most common pathogenic avian clones. They have 2.236 Mb of the ~2.4 Mb genome as four contigs and expect to complete the project very soon. 76% of ORFs have very close homologues in the *H. influenzae* genome and many have *E. coli* matches. There is a lack of colinearity, though; the gene order seems very jumbled up. They have already identified several genes of interest in determining pathogenicity and have carried out further study of several by producing a quadruplicated glass slide microrarray and comparing the levels of expression of these genes in virulent and avirulent strains. One gene in particular showed significant expression in all virulent isolates and none in avirulent ones. They plan to sequence more strains from other hosts in an attempt to identify the differences between the different host specific strains.

Bruce Nicholson (University of Maine, USA) reported on the results of **comparative studies of aquatic birnaviruses**. These viruses have small double-stranded RNA genomes which comprise five ORFs. An amino acid comparison between a large number of strains identified one area of hypervariation and a few regions of lower variation. Most of the residues, however, show very low variation. There are some amino acid variations that are geographically region-specific. The most divergent strain had 19% amino acid difference. In the majority of cases, the genotype difference correlated well with serogroup profiles. They found only eight amino acids that differed between high virulence and low virulence strains, all of which were located in the largest gene.

Cathy Costello (Boston University, USA) discussed the application of **mass spectrometry** to the analysis of proteins. MALDI-TOF has been applied to 300 000 MW collagen from chicken and also to a tryptic digest of the alpha chain. Her group are testing a matrix with trypsin already bound on it, allowing them to digest the sample on the matrix. The method can detect post-translationally modified forms of proteins, such as proteins with Cys–Cys linkages and carbohydrate attachments. It is sometimes possible to tell which carbohydrate group has been added. By seeing where the charges that are added during the mass spectrometry go, they can tell which amino acids are on the surface of a protein. Some proteins can even be sequenced using this technology, depending upon their molecular weight.

Ross Overbeek (Integrated Genomics, USA) spoke about the application of **comparative mapping** to the interpretation of **microbial genomes**. His company has access to approximately sixty five 95%-sequenced genomes. They have found that completion of the last 5% of any genome (usually gap closing) costs almost as much as sequencing the first 95%. So, since their goal is gene identification rather than completed genomes, they stop at 95%. They have been using WIT, an environment for comparative genomics (**http://igweb.integratedgenomics.com/IGwit/**), to perform their analyses. First, they looked at how many genes from metabolic pathways occurred close to another gene from the same pathway in microbe genomes. They found that this occurred for about 35–55% of the genes analysed and they found 122 conserved clusters between *E. coli* and *B. subtilis*. Taking two less related microbes, for example *V. cholerae* and *E. coli*, they looked for close bi-directional gene matches in stretches with ~300 bp between genes. If these matches could be found in other genomes, this raised the significance that the genes are linked. In this way, a conserved orphan gene consistently found within a region of genes from the same pathway can be putatively assigned to that pathway. They also feel that by looking at more closely related microbes, this analysis could be extended to the search for regulatory sequences.

Copyright © 2000 John Wiley & Sons, Ltd.

Robin Buell (TIGR, USA) presented the tools and services publicly available at the **TIGR website**. They have been doing a lot of work on annotation of genomes. They use Glimmer to identify ORFs and use homology searches, motif searches and regulatory sequence searches to assign putative identities. They also look for frame shifts and ambiguities and search for repeats. They use the same system for each genome to faciliate comparative studies; this has worked well with the 10 microbial genomes that they have completely sequenced. This is an approach that they are advocating for the *Arabidopsis* data. They plan to download all of the five chromosome sequences and, after producing the unified annotation, to provide search engines and other tools. They also provide a list of all on-going genome sequencing projects with links to searchable archives of the data. TIGR is also involved in post-genome studies such as mutagenesis projects, and they plan to have data from experimental approaches, such as global gene expression analyses, and from *in silico* studies, such as comparative mapping and structure prediction tools available on the site. They also maintain gene indices for partial genomes, including rice and zebrafish, in which all ESTs are checked for overlaps in order to produce consensus sequences that give better representations of genes. These indices can be searched with DNA or amino acid sequences, or using keyword or gene or clone name queries.

Mike Sadowsky (University of Minnesota, USA) described work on microbial plasmids aimed at discovering which genes are responsible for **degradation of herbicides and pesticides**. First they identified a pseudomonad that could degrade a herbicide, Atrazine. Then, by transforming another strain with cloned genome fragments, they identified an Atrazine dechlorinase gene and a second gene, located 7 kb away, which was adding a hydroxyl group to the product of the dechlorinase. By looking for these genes in other genera, they found that they were very common and reasoned that they could have been transferred by plasmids. Mating with *E. coli* caused transfer of the enzyme activity, proving that it was located on a plasmid. Isolation and sequencing of the 100 kb plasmid uncovered a third atrazine degradation gene and an incomplete set of mercury degradation genes. The Atrazine degradation genes were shown to be flanked by insertion sequences. These are most likely responsible for the movement of the genes into some microbial genomes.

Martin Rosenberg (SKB, USA) spoke about the application of genomics to **antimicrobial discovery**. Between SKB and Incyte, there are now ~70 near-complete microbial genomes, and often they have sequenced two or three independent clinical isolates of a particular microbe. They have started by looking at microbial genes with no homologues or poor homologues in host genomes. A classical example of this would be the amino acyl-tRNA synthetase genes, which are essential. There are 19 of them in Gram-positive bacteria, one of which, for aatRNAIle, is already the target of a very good antibiotic. So, they explored the other 18 as potential targets. So far they have three good lead compounds that work well against a range of microbes and have a predicted selectivity of greater than 1000-fold (for action against microbes compared to against mammalian cells). Using a gel-based system they have performed expression analysis studies on some 2000 *Staphylococcus aureus* and *Streptomyces pneumoniae* genes. By multiplexing the samples in each lane, they can do one experiment on just three gels and they have shown that it can be done using *in vitro* samples or infected tissue. They are currently comparing different times of infection, with a particular interest in the late symptomatic phase, when patients present themselves for treatment. They have gone on to delete several of the genes identified as being highly expressed in the bacteria during this phase. Around 30% were essential genes and about 45% caused significant attenuation upon deletion. They have also looked at signal transduction genes, in particular those that have been shown to be essential in some strains. They have found one new essential gene and seven which, when deleted in *S. pneumoniae* result in highly attenuated microbes; however, it appears that essentiality is not always conserved.

David Hopwood (John Innes Centre) reported on the sequencing of the ***Streptomyces coelicolor*** genome, which is nearing completion. This bacterium produces a blue antibiotic (hence the name), amongst others and is responsible for ~80% of the actinomycete derived antibiotics on the market. It has an 8 Mb linear genome with a central origin of replication. The genome is replicated bidirectionally; the Okazaki strand is finished with the aid of a protein which acts as an inward primer on the 3′

Copyright © 2000 John Wiley & Sons, Ltd.

end. An ordered contig of cosmids has been constructed; this is being used as the template for sequencing, at the Sanger Centre. The high GC ratio of this genome results in a low frequency of random stop codons, thus there are many potential ORFs; however, coding sequence differs from non-coding sequence in the third base GC ratio, making ORFs stand out. They are predicting 7400 genes, giving a gene density of 1.08 kb per gene. Although it is possible to delete $\sim$1 Mb from the ends of the chromosome without loss of viability, the gene density in these regions is not reduced; it seems that essential genes are only found towards the centre of the chromosome. The genes found at the chromosome ends are often for metabolism of less common substrates and would only be needed under certain conditions. There does not appear to be any evidence of an ancient genome duplication such as that seen in yeast. A large number of the genes appear to be used for antibiotic synthesis and sporulation functions, both of which have complex patterns of regulation. *S. coelicolor* has many more ABC transporter genes and transciptional regulators than in *E. coli* and many genes used for environmental sensing, which could reflect the complexity of the soil environment it inhabits.

Adolphus Van Loon (Hoffmann LaRoche, Switzerland) gave a presentation on their work towards the **production of a microbial phytase** for animal feeding. Feeding farm animals with this enzyme would enable them to utilize organic phosphate in the food, rather than needing the addition of inorganic phosphate. Using a comparison of fungal phytases they defined a consensus sequence, this enzyme was then expressed in *Hansenula polymorpha* (which secretes the protein) and tested for pH optimum, activity and heat stability. It performed better in all three tests than any native phytase. They plan to sequence more fungal phytases to improve the protein further towards their ideal profile of highest activity at animal stomach pH, resistance to the high temperature that food pellets must be exposed to before use and high overall activity.

Mary Lidstrom (University of Washington, USA) spoke about the efforts of her group to analyse the genome of the **plant epiphyte *Methylobacterium extorquens***. These are the dominant plant leaf epiphytes and are responsible for the pink bacterial footprint that leaves make on agar plates. They use the methanol that plants excrete through the stomata and produce zeatin, a plant growth hormone. Their first, 1X coverage random sequencing yielded about 60% of the 6 Mb genome. This sequence will be expanded in the second 5–6X coverage phase. So far, they have found new assimilation genes, new transcriptional regulators genes involved in PHB synthesis and three new formate dehydrogenases. Many of these genes have been deleted to check for phenotypes matching their predicted roles, with some success. They have found some genes that are most like plant genes and some which are more closely related to genes from plant symbionts and plant pathogens. They have also identified a long stretch of genes most closely related to archaeal genes. It is unclear as yet if these have been acquired by lateral transfer or if they truly have a role in this organism.

Eric Triplett (University of Wisconsin, USA) reported on the results of studies in which bacteria are modified to function better in roles valuable to agriculture. **Rhizobia** live in nodules in the roots of plants such as soybean and alfalfa and fix nitrogen, enabling the plant to use this food source. His group engineered a plasmid containing genes for production of and resistance to a particular antibiotic, along with a locus involved in nitrogen fixation that had been shown to increase biomass in plants. Rhizobium strains carrying this plasmid could out-compete natural strains and caused the plants to grow better. They have extended this work to a diazotrophic *Acetobacter* that they identified as the reason why certain lines of sugarcane were found not to need nitrogen-based fertilizers. Although they showed that it is these bacteria that are fixing nitrogen for the plant, they were unable to get them to do the same job for maize, since the root environment is different. There is not enough carbon source in the maize roots for these bacteria to fix nitrogen.

### Summary

The Plant and Animal Genomes meeting covers an enormous range of species and hence is very difficult to attend as a delegate with a general interest, since many workshops take place at the same time. However, the posters were all together in one hall, so it was possible for people to get a look at what is going on with related species during the poster sessions. An amazing amount of comparative

Copyright © 2000 John Wiley & Sons, Ltd.

mapping is under way in plant, animal and microbe genome studies. Many of the talks detailed in this review clearly show the immense value to be gained from the application of this technique. As can be expected, the application of functional genomics is less common, particularly in the animal genomes, where the emphasis is still on genetic and physical mapping and sequencing. In the plant genomes, however, several forays into expression analysis and proteomics were presented and are detailed here, in fact in some cases, the plant genomes are so intractable that these techniques may well be the best way forward. Although the bias was towards organisms of agricultural importance, several technologies of interest to all communities were detailed at the meeting and are described in this review. The improvements reported in SNP detection and typing technologies in particular will be of interest to the mammalian genome researchers.

Many of the results and methodologies presented at the Agricultural Microbes meeting have clear applications in agriculture or medicine. Several of the teams working on these projects have an excellent supply of related genome sequences to mine and they are already starting to take advantage of functional genomics approaches, such as microarray expression analysis. This first meeting was well received by all delegates and I am sure that the number of applicants will rise next year.

---

The Meeting Highlights of *Comparative and Functional Genomics* aim to present a commentary on the topical issues in genomics studies presented at a conference. The Meeting Highlights are invited. Each represents a personal critical analysis of the current reports and aims to provide implications for future genomics studies.

This article was written by Dr Joanne Wixon (Managing Editor).

*The* **Genomics** *Website at Wiley*

**www.wiley.co.uk/genomics**

The **Genomics** website at Wiley is a new and **DYNAMIC** resource for the genomics community, offering **FREE** special feature articles and new information **EACH MONTH**.

Find out more about *Comparative and Functional Genomics*, and enter the **Free Prize Draw** for the chance to win a year's free subscription.

Visit the **Library** for hot books in Genomics, Bioinformatics, Molecular Genetics and more.

Click on **Primary Research** for information on all our up-to-the minute journals, including: *Genesis*, *Bioessays, Gene Function and Disease*, and the *Journal of Gene Medicine*.

Let the **Genomics** website at Wiley be your **guide** to genomics-related web sites, manufacturers and suppliers, and a calendar of conferences.

WILEY

1516

Copyright © 2000 John Wiley & Sons, Ltd. 

